# Stability of Anthocyanins from Red Grape Skins under Pressurized Liquid Extraction and Ultrasound-Assisted Extraction Conditions

**DOI:** 10.3390/molecules191221034

**Published:** 2014-12-15

**Authors:** Ali Liazid, Gerardo F. Barbero, Latifa Azaroual, Miguel Palma, Carmelo G. Barroso

**Affiliations:** 1 Department of Analytical Chemistry, University of Cadiz, Puerto Real 11510, Spain; E-Mails: ali.liazid@uca.es (A.L.); gerardo.fernandez@uca.es (G.F.B); carmelo.garcia@uca.es (C.G.B.); 2 Faculty of Sciences, Abdelmalek Essaâdi University, Tetouan 93000, Morocco; E-Mail: latifaazaroual@gmail.com

**Keywords:** anthocyanins, stability, pressurized liquid extraction, ultrasound-assisted extraction, red grape skin

## Abstract

The stability of anthocyanins from grape skins after applying different extraction techniques has been determined. The following compounds, previously extracted from real samples, were assessed: delphinidin 3-glucoside, cyanidin 3-glucoside, petunidin 3-glucoside, peonidin 3-glucoside, malvidin 3-glucoside, peonidin 3-acetylglucoside, malvidin 3-acetylglucoside, malvidin 3-caffeoylglucoside, petunidin 3-*p*-coumaroylglucoside and malvidin 3-*p*-coumaroylglucoside (*trans*). The techniques used were ultrasound-assisted extraction and pressurized liquid extraction. In ultrasound-assisted extraction, temperatures up to 75 °C can be applied without degradation of the aforementioned compounds. In pressurized liquid extraction the anthocyanins were found to be stable up to 100 °C. The relative stabilities of both the glycosidic and acylated forms were evaluated. Acylated derivatives were more stable than non-acylated forms. The differences between the two groups of compounds became more marked on working at higher temperatures and on using extraction techniques with higher levels of oxygen in the extraction media.

## 1. Introduction

The anthocyanins are a group of compounds that belong to the flavonoid family and these are of great interest in the food industry, mainly due to their colouring properties [1,2]. However, interest in these compounds has increased in recent years due to their antioxidant, anti-inflammatory, antiviral, antibacterial and even anticarcinogenic properties [3,4,5].

Anthocyanins are found in many plants but red grapes are of particular interest due to the presence of high concentrations of a wide variety of these compounds. The stability of anthocyanins is dependent on various factors, such as the structure, whether or not they are bound to sugars, the pH, light, the presence of ions and enzymes and, most importantly, temperature [6]. The levels of these compounds obtained from plants are often influenced by the conditions used for their extraction.

Various techniques have been proposed for the extraction of anthocyanins and these involve the use of methanol, ethanol, acetone, water, or mixtures of these solvents [7]. The addition of small quantities of hydrochloric or formic acid has been used as a way to improve the extraction outcome [8]. The techniques used for extraction from semi-solid or viscous samples (such as grape must) have mainly involved extraction by maceration with solvents [9], followed by liquid-liquid extraction or the use of solid phase extraction (SPE) as a step for cleaning and/or concentration of the extract. In any case, such techniques require long extraction times and have limited efficiency.

For the reasons outlined above, new and more efficient techniques are currently being used that enable a reduction in both the extraction time and the consumption of organic solvents. Pressurized Liquid Extraction (PLE) and Ultrasound-Assisted Extraction (UAE) are two techniques that can meet these requirements. In these cases, the first step should be to test the stability of the compounds to be extracted at different extraction temperatures used in these techniques in order to determine a reliable working range.

UAE has been used in the extraction of organic compounds from soil, plant tissues and packing materials [10]. Ultrasound has a mechanical effect that enables greater penetration of the solvents into the matrix and increases the surface contact between the solid and the liquid. In addition, the occurrence of cavitation leads to cell breakage and this can increase the speed of extraction. An application of UAE for the extraction of anthocyanins from red raspberries was found in a review of the literature [11]; however, studies on the stability of these compounds during the extraction process were not found.

PLE is a technique that is used to prepare samples when pressure and temperature are the main extraction variables in the design of fast methods for extraction from solid or semi-solid materials [12]. Pressure is used to increase the contact between the liquid and the sample and also to maintain the solvent in a liquid state when working at temperatures above its boiling point [13]. At these temperatures, breakage of the analyte-matrix bonds is facilitated. Additionally, the temperature can have a significant effect on the properties of the solvent and may lead to a change in its dielectric constant, thereby affecting the selectivity of the extraction. Applications of PLE for the determination of anthocyanins in grapes were not found in our review of the literature.

The aim of the study described here was to determine the effects of various possible extraction temperatures on the stability of anthocyanins from grape skins. UAE and PLE working conditions were therefore assessed using anthocyanins previously extracted from grape skins. The results were compared with those obtained previously on using microwave-assisted extraction (MAE).

## 2. Results and Discussion

For each extraction technique studied, a range of different temperatures was evaluated according to the working range available. It must be noted that extraction processes were not run because a solid matrix was not used, rather the investigation was carried out on liquid samples, *i.e.*, a standardized extract. The standardized extract was prepared using grape skins as the solid material and methanol as the solvent under very mild extraction conditions. The solid material contained all types of anthocyanins previously determined in grape skins. UAE and PLE conditions were applied to the standardized extracts at different working temperatures. The experiments were run in order to assess the stability of both glucosylated anthocyanins and their derivatives in the sample under given extraction conditions. Therefore, full recovery would be expected if degradation processes did not occur under the working conditions. On the other hand, recoveries of less than 100% with reference to the starting values in the extract would indicate degradation of anthocyanins during the experiments. All experiments were evaluated statistically in order to establish reliable results.

Temperatures above 75 °C were not studied in the ultrasound-assisted extraction as this would lead to a low level of reproducibility, principally due to the evaporation of water. As a consequence, it was considered that higher temperatures would not be applicable in practice, even if the anthocyanins were stable under such conditions. As far as PLE is concerned, temperatures were studied up to the point where clear degradation of the anthocyanins was found.

For each technique, the standardized extract from the grape skins was used as a reference at the same dilution as developed for each extraction technique. Two references were processed, one at the beginning and one at the end of each day on which the extractions were carried out with each technique. The average of the chromatographic areas of each of the peaks was obtained from the chromatogram and a value of 100 was assigned to the mean area of each.

One of the most likely degradation processes to occur under the extraction conditions is the breakage of the bonds between the anthocyanins and the different acids to which they are bound. As a consequence, the effects of the extractions on the anthocyanins under investigation were divided into two groups for evaluation: (i) glucosylated compounds and (ii) acylated derivatives of these compounds.

### 2.1. Ultrasound-Assisted Extraction

**Figure 1:**
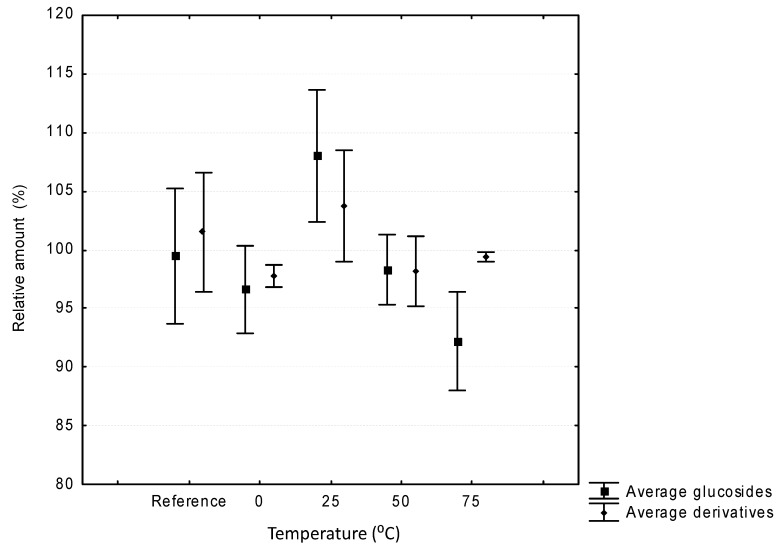
Average recoveries (mean ± standard deviation) of anthocyanins at different working temperatures (0, 25, 50 and 75 °C) and the reference in the ultrasound-assisted extraction system.

Extraction temperatures between 0 °C and 75 °C were evaluated. The presence of methanol as the solvent for the standardized extract prevented freezing of the sample during the extraction process at 0 °C. Temperatures above 75 °C were also assayed but, due to the high variability in the results and the need for the addition of further solvent during the experiment, these higher temperatures were not taken into account when analyzing the results. The average recoveries of the compounds quantified in the chromatograms are shown in Figure 1.

No statistically significant differences were found between the results obtained at the different temperatures studied. This finding implies that both the glucosylated compounds and their derivatives are stable under the conditions used in the ultrasound extraction up to 75 °C. Similar results were obtained for other phenolics [14], but results on the stability of anthocyanins under UAE working conditions have not been reported previously.

### 2.2. Pressurized Liquid Extraction

**Figure 2:**
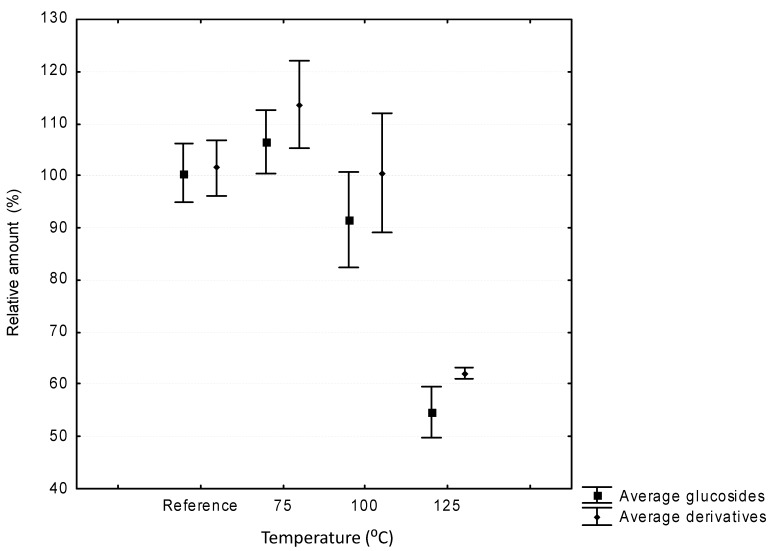
Average recoveries (mean ± standard deviation) of anthocyanins at different working temperatures (75, 100 and 125 °C) and reference sample in the pressurized liquid extraction system.

The PLE technique was used at temperatures from 75 to 125 °C. Lower temperatures were not tested because the aim of the work was to find methods that could, where appropriate, produce faster extractions than the more commonly used methods, meaning that it was essential that high extraction temperatures were used. The results, which are grouped into compound families, are shown in Figure 2. It can be seen from Figure 2 that both the glucosylated anthocyanins and their acylated derivatives are stable up to 100 °C. This means that extractions can be carried out up to this temperature without having an adverse effect on the stability of the anthocyanins. It must also be noted that significant differences were not found between the glucosides and their acylated derivatives in terms of their behaviour under these extraction conditions.

On working at temperatures around 100 °C dramatic degradation was observed for both glucosides and their acylated derivatives. Degradation levels between 40% and 50% were reached. The degradation observed at 125 °C is probably the result of oxidation reactions promoted by the very high temperature. It should be noted that conversion from acyl derivatives to the glucosyl forms was not observed. This result indicates that PLE would produce unreliable results if the system was operated above 100 °C for the extraction of anthocyanins.

### 2.3. Effects on Individual Anthocyanins

In order to develop extraction methods for specific anthocyanins present in the grape extract, it would be of interest to determine the sensitivity to degradation of compounds on an individual basis, rather than globally, and for this reason an attempt was made to determine the differences between the different compounds analyzed. In this respect, significant differences have been described in the literature for related compounds.

The influence of the specific functional groups in the chemical structure on the stability during extractions can also be obtained. This information would be of interest when estimating the stability of other related compounds.

**Figure 3:**
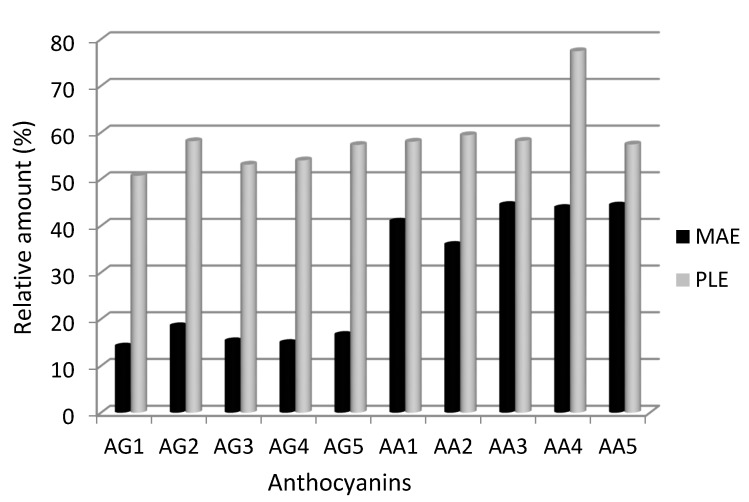
Recoveries of anthocyanins at 125 °C using microwave-assisted extraction and pressurized liquid extraction conditions. AG1–AG5: glycosylated anthocyanins, AA1–AA5 acylated anthocyanin derivatives. See Table 1 for a full identification of anthocyanins.

In order to achieve this goal, an analysis was carried out on the results obtained in two different systems, PLE and MAE, carried out at the same temperature and where partial degradation of the anthocyanins occurred, namely 125 °C. The MAE system was selected because the extraction under these conditions can be run at the same temperature as PLE but with a higher level of oxygen in the medium. This study would enable the effects of high oxygen levels to be evaluated. The results obtained are shown in Figure 3 and all of these compounds gave values that are significantly lower than that of the reference.

**Table 1:** Amounts of anthocyanins in the standardized extract.

Anthocyanin	mg/L *
Delphinidin 3-glucoside (AG1)	26.8
Cyanidin 3-glucoside (AG2)	11.2
Petunidin 3-glucoside (AG3)	36.1
Peonidin 3-glucoside (AG4)	130.0
Malvidin 3-glucoside (AG5)	381.2
Peonidin 3-acetylglucoside (AA1)	14.5
Malvidin 3-acetylglucoside (AA2)	46.4
Malvidin 3-caffeoylglucoside (AA3)	7.9
Petunidin 3- *p-*coumaroylglucoside (AA4)	7.1
Malvidin 3- *p*-coumaroylglucoside (*trans*) (AA5)	61.6

*: Expressed as malvidin-3-glucoside.

Comparison of the five glycosylated compounds (AG1–AG5) shows that, regardless of their chemical structure, *i.e*., the ring substituents, their sensitivity to degradation is practically the same both under the high oxidation (MAE at 125 °C) and the low oxidation (PLE 125 °C) conditions. On the other hand, acylated derivatives of the glucosides were generally found to be more stable than the glucosides themselves when the more oxidative conditions were used (MAE 125 °C), whereas the degradation percentages were similar when the only factor favourable to degradation was temperature. In any case, as in the case of glucosides, differences were not observed between the different susceptibilities to degradation for the five acyl derivatives studied individually, thus showing that this behaviour is also independent of chemical substitution in the aromatic ring. The nature of the chemical substituents in the aromatic rings does not influence the stability of anthocyanins during the extraction process, *i.e.*, the maximum extraction temperatures found in this study could be applied to other anthocyanins present in different samples even in cases where different functional groups are present in the aromatic rings.

## 3. Experimental Section

### 3.1. Samples

The grape skins were obtained from red grapes of the Tintilla de Rota variety grown in Jerez (Spain). Levels for anthocyanins in the grape skins (mg Kg^−1^ FW) were as it follows: delphinidin 3-glucoside: 1.26; cyanidin 3-glucoside: 0.87; petunidin 3-glucoside: 2.31; peonidin 3-glucoside: 4.33; malvidin 3-glucoside: 16.99; peonidin 3-acetylglucoside: 1.21; malvidin 3-acetylglucoside: 5.52; malvidin 3-caffeoylglucoside: 0.66; petunidin 3-*p*-coumaroylglucoside: 1.96; malvidin 3-*p*-coumaroylglucoside (*trans*): 12.46. The skins were separated from the seeds and pomace, and then milled in a coffee grinder for 2 min, in bursts of 15 s in order to avoid sample heating. The sample was stored at –20 °C prior to extraction. A standardized extract with a known anthocyanin concentration was used in order to evaluate the stability accurately. This extract was prepared because standards for most of the anthocyanins were not available and, as a consequence, they had to be obtained from real samples in order to assess their stabilities. This extract was obtained by solid-liquid extraction of the ground grape skin in an ultrasonic bath. In order to obtain a sufficient amount of extract, approximately 100 g of ground grape skin was steeped in 250 mL of 100% methanol for 30 min at 25 °C. Re-extractions of the solid sample were carried out. Extractions obtained using this protocol provided approximately 1 L of grape skin extract. The extract was concentrated using a nitrogen stream at 40 °C. It was not dried but used in liquid form. The resulting concentrated extract was stored at –20 °C until it was used in the stability studies. Concentrations of anthocyanins in the concentrated extract are shown in Table 1. Values are expressed as malvidin-3-glucoside equivalents because of most standards were not commercially available. Identification for those compounds were previously developed by the authors by HPLC-MS [15].

### 3.2. Chemicals and Solvents

All of the reagents used were of analytical grade: methanol and formic acid were obtained from Merck (Darmstadt, Germany). HPLC grade water was obtained from a Milli-Q system (Millipore, Bedford, MA, USA). All samples were filtered through a 0.45 µm nylon syringe filter (Millipore) before chromatographic analysis. All extractions were performed in triplicate.

### 3.3. Ultrasound-Assisted Extraction

UAE extraction conditions were applied in a water bath at 400 W (J.P. Selecta, Barcelona, Spain). The extraction protocol was carried out on 1.5 g of the standardized grape skin extract in approximately 9 mL of water for 20 min. The experiments were performed at constant temperature by means of a temperature controller coupled to the ultrasonic bath. Four temperatures were assayed: 0, 25, 50 and 75 °C. After each extraction, the volume of extract was made up to 10 mL with water.

### 3.4. Pressurized Liquid Extraction

Extraction conditions were applied in a Dionex ASE 200 extractor (Dionex, Sunnyvale, CA, USA). The standardized grape skin extract (8 g) was mixed with sea sand (Panreac, Barcelona, Spain) and placed in an 11 mL stainless steel extraction cell. A cellulose filter (Dionex, Sunnyvale, CA, USA) was placed at the bottom of the extraction cell. Nitrogen was used to purge and dry the extraction chambers during the extractions.

The extraction chamber was filled with water, pressurized to 100 atm, and heated to temperatures ranging from 75 to 150 °C for 20 min. The extracts were topped up to 50 mL with water, and these samples were then analyzed by RP-HPLC.

### 3.5. Ultra-Performance Liquid Chromatography (UPLC)

**Figure 4:**
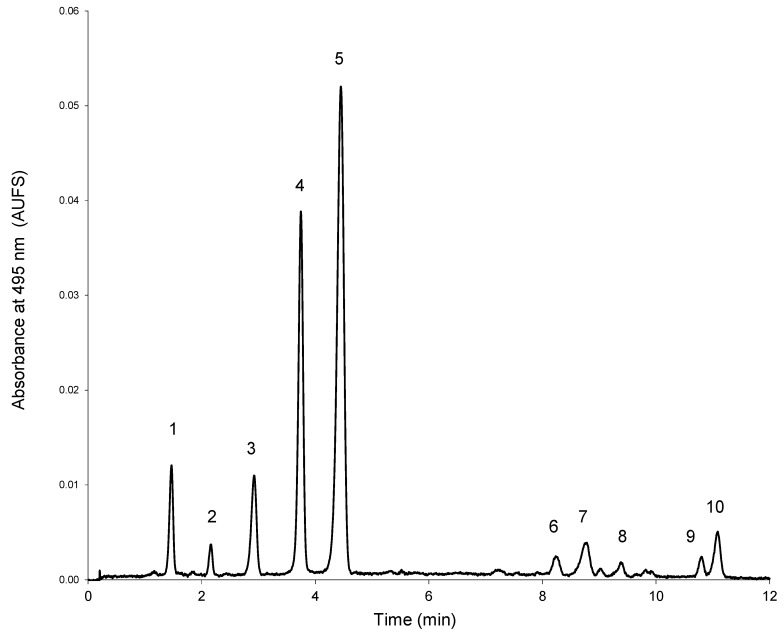
Typical chromatogram of the standardized extract. **1**: Delphinidin 3-glucoside; **2**: cyanidin 3-glucoside; **3**: petunidin 3-glucoside; **4**: peonidin 3-glucoside; **5**: malvidin 3-glucoside; **6**: peonidin 3-acetylglucoside; **7**: malvidin 3-acetylglucoside; **8**: malvidin 3-caffeoylglucoside; **9**: petunidin 3-*p*-coumaroylglucoside; **10**: malvidin 3-*p*-coumaroyl-glucoside (*trans*).

UPLC analyses were performed on a Waters Acquity Ultra Performance Liquid Chromatographic system (Waters, Milford, MA, USA) equipped with a PDA detector, an autosampler and a column oven to control the temperature of the analytical column (35 °C). Data were collected and processed by Empower chromatographic software (Waters). Chromatographic separation was achieved in an Acquity UPLC BEH C18 column (100 mm × 2.1 mm, 1.7 µm, Milford, MA, USA) equipped with an in-line 0.2 µm Acquity filter. Mobile phase A was 5% formic acid in water and mobile phase B was methanol. The gradient was as follows: 0 min, 5% B; 11 min, 29.6% B; 12 min, 30% B; 12.5 min, 100% B. A flow of 0.7 mL·min^−1^ was used. A typical chromatogram of the diluted standardized extract is shown in Figure 4.

## 4. Conclusions

The stabilities of anthocyanins under the working conditions used for UAE and PLE were specifically studied for the first time. In view of the results, all assayed anthocyanins showed full stability under the UAE conditions, including working temperatures from 0 °C to 75 °C. It is therefore possible to employ extraction methods based on UAE up to 75 °C without degradation of the components.

The high temperatures usually applied in PLE methods led to significant degradation of anthocyanins. Specifically, the use of temperatures above 100 °C led to 50% degradation for most anthocyanins. It is therefore recommended that PLE for anthocyanins should only be employed up to 100 °C.

The glucosyl anthocyanins proved to be more susceptible to degradation than the acyl derivatives, with the difference in susceptibility to degradation being greater on working at higher temperature and in the presence of greater levels of oxygen. The results show that, depending on the specific compounds present in the samples (glucosides or acyl derivatives), PLE or UAE can be selected as the extraction method and the stability of anthocyanins would be guaranteed during the extraction process.

The stabilities of individual anthocyanins were also assessed and significant differences were not found with respect to the susceptibility to degradation for the studied compounds. Therefore, a relationship between the type of substituent present in either glycosylated or acyl derivatives and the susceptibility to degradation was not established. This means that information about stability of compounds beyond the scope of this study cannot be extrapolated from the results obtained on the assayed compounds.

## References

[1] De Carvalho Alves A.P., Correa A.D., Marques Pinheiro A.C., de Oliveira F.C. (2013). Flour and anthocyanin extracts of jaboticaba skins used as a natural dye in yogurt. Int. J. Food Sci. Technol..

[2] Pina F., Melo M.J., Laia C.A.T., Parola A.J., Lima J.C. (2012). Chemistry and applications of flavylium compounds: A handful of colours. Chem. Soc. Rev..

[3] Kruger M.J., Davies N., Myburgh K.H., Lecour S. (2014). Proanthocyanidins, anthocyanins and cardiovascular diseases. Food Res. Int..

[4] Martin Bueno J., Saez-Plaza P., Ramos-Escudero F., Maria Jimenez A., Fett R., Asuero A.G. (2012). Analysis and Antioxidant Capacity of Anthocyanin Pigments. Part II: Chemical Structure, Color, and Intake of Anthocyanins. Crit. Rev. Anal. Chem..

[5] Pojer E., Mattivi F., Johnson D., Stockley C.S. (2013). The Case for Anthocyanin Consumption to Promote Human Health: A Review. Compr. Rev. Food Sci. Food Saf..

[6] Sui X., Dong X., Zhou W. (2014). Combined effect of pH and high temperature on the stability and antioxidant capacity of two anthocyanins in aqueous solution. Food Chem..

[7] Welch C.R., Wu Q., Simon J.E. (2008). Recent advances in anthocyanin analysis and characterization. Curr. Anal. Chem..

[8] Turker N., Erdogdu F. (2006). Effects of pH and temperature of extraction medium on effective diffusion coefficient of anthocynanin pigments of black carrot (*Daucus carota* var. L.). J. Food Eng..

[9] Karvela E., Makris D.P., Kalogeropoulos N., Karathanos V.T. (2009). Deployment of response surface methodology to optimise recovery of grape (*Vitis vinifera*) stem polyphenols. Talanta.

[10] Barbero G.F., Liazid A., Palma M., Barroso C.G. (2008). Ultrasound-assisted extraction of capsaicinoids from peppers. Talanta.

[11] Chen F., Sun Y., Zhao G., Liao X., Hu X., Wu J., Wang Z. (2007). Optimization of ultrasound-assisted extraction of anthocyanins in red raspberries and identification of anthocyanins in extract using high-performance liquid chromatography-mass spectrometry. Ultrason. Sonochem..

[12] Mendiola J.A., Herrero M., Cifuentes A., Ibañez E. (2007). Use of compressed fluids for sample preparation: Food applications. J. Chromatogr. A.

[13] Carabias-Martinez R., Rodriguez-Gonzalo E., Revilla-Ruiz P., Hernandez-Mendez J. (2005). Pressurized liquid extraction in the analysis of food and biological samples. J. Chromatogr. A.

[14] Palma M., Piñeiro Z., Barroso C.G. (2001). Stability of phenolic compounds during extraction with superheated solvents. J. Chromatogr. A.

[15] Guerrero R.F., Liazid A., Palma M., Puertas B., González-Barrio R., Gil-Izquierdo A., Barroso C.G., Cantos-Villar E. (2009). Phenolic characterisation of red grapes autochthonous to Andalusia. Food Chem..

